# Local anesthetic bupivacaine induced ovarian and prostate cancer apoptotic cell death and underlying mechanisms *in vitro*

**DOI:** 10.1038/srep26277

**Published:** 2016-05-19

**Authors:** Wei Xuan, Hailin Zhao, James Hankin, Lin Chen, Shanglong Yao, Daqing Ma

**Affiliations:** 1 Department of Anesthesiology, Renji Hospital, School of Medicine, Shanghai Jiaotong University, Shanghai 200001, China; 2 Institute of Anesthesiology and Critical Care Medicine, Union Hospital, Tongji Medical College, Huazhoung University of Science and Technology, Wuhan, Hubei, China; 3 Anaesthetics, Pain Medicine and Intensive Care, Department of Surgery and Cancer, Faculty of Medicine, Imperial College London, Chelsea & Westminster Hospital, London, UK

## Abstract

Retrospective studies indicate that the use of regional anesthesia can reduce cancer recurrence after surgery which could be due to ranging from immune function preservation to direct molecular mechanisms. This study was to investigate the effects of bupivacaine on ovarian and prostate cancer cell biology and the underlying molecular mechanisms. Cell viability, proliferation and migration of ovarian carcinoma (SKOV-3) and prostate carcinoma (PC-3) were examined following treatment with bupivacaine. Cleaved caspase 3, 8 and 9, and GSK-3β, pGSK-3β^tyr216^ and pGSK-3β^ser9^ expression were assessed by immunofluorescence. FAS ligand neutralization, caspase and GSK-3 inhibitors and GSK-3β siRNA were applied to further explore underlying mechanisms. Clinically relevant concentrations of bupivacaine reduced cell viability and inhibited cellular proliferation and migration in both cell lines. Caspase 8 and 9 inhibition generated partial cell death reversal in SKOV-3, whilst only caspase 9 was effective in PC-3. Bupivacaine increased the phosphorylation of GSK-3β^Tyr216^ in SKOV-3 but without measurable effect in PC3. GSK-3β inhibition and siRNA gene knockdown decreased bupivacaine induced cell death in SKOV-3 but not in PC3. Our data suggests that bupivacaine has direct ‘anti-cancer’ properties through the activation of intrinsic and extrinsic apoptotic pathways in ovarian cancer but only the intrinsic pathway in prostate cancer.

Cancer recurrence and metastasis are significant causes of death in cancer patients^1^. Surgical resection of solid tumors can be curative. However, surgery itself inducing stress responses is immunosuppressive and the inadvertent seeding of cancer cells may also occur during an operation. This increases the risk of tumor metastasis during the perioperative period^2^,^3^.

During surgery, local/regional anesthesia (LA/RA) techniques are used for various reasons in cancer patients. These can range from pain management to decrease opioid use^4^,^5^,^6^. In light of the potential benefits of LA/RA use in cancer patients, there has been an increased focus on investigating the mechanisms involved^7^. Retrospective studies indicate that the use of LA/RA decreases the risk of metastasis, cancer recurrence, and improves overall survival^8^,^9^. More specifically and relevant to this study, decreased cancer recurrence has been reported with the use of epidural anesthesia in ovarian and prostate carcinomas^10^,^11^.

There is a strong association between the use of LA/RA and the preservation of cell mediated immunity surgical stress response modulation^12^. Recent *in vitro* studies have examined the underlying molecular mechanisms of local anesthetics and cancer cell biology^13^,^14^. Despite this progress, knowledge of potential direct mechanisms is limited.

The aim of this study is to investigate the effects of the commonly used local anesthetic bupivacaine on the viability, proliferation and migration properties of human ovarian carcinoma and prostate carcinoma cell lines. Furthermore, bupivacaine induced cancer cell death and potential underlying molecular mechanisms are explored. A novel approach is utilized, with a focus on the activity of glycogen synthase kinase-3β (GSK-3β), a multifunctional enzyme involved in numerous cellular processes. We investigated its potential interactions with bupivacaine on cancer cell biology. In this context, the phosphorylation activity of GSK-3β’s residues of tyrosine (active form) or serine (inactive form)^15^ was investigated.

## Results

### Bupivacaine on cancer cell viability and chemotherapy sensitivity

**Figure 1:**
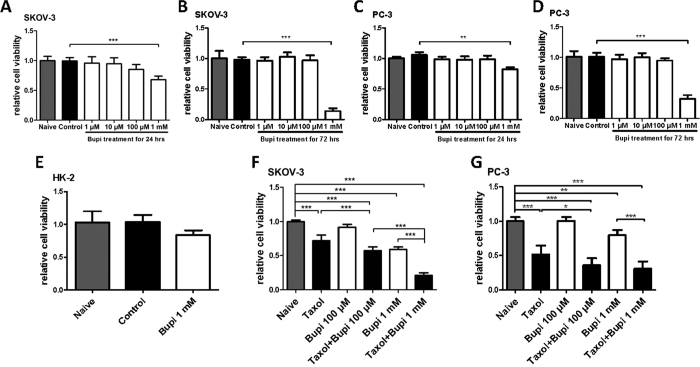
Bupivacaine alone and combined with anti-cancer drug decreased viability of both ovarian cancer (SKOV-3) and prostate cancer (PC-3) cells. SKOV-3 and PC-3 cells were treated with bupivacaine (Bupi) from 1 μM to 1 mM for 24 or 72 h and cell survival was assessed by 3-(4,5-dimethylthiazol-2-yl)-2,5-diphenyltetrazolium bromide (MTT) assay. (**A**) SKOV-3 cells with bupivacaine for 24 h. (**B**) SKOV-3 cells with bupivacaine for 72 h. (**C**) PC-3 cells with bupivacaine for 24 h. (**D**) PC-3 cells with bupivacaine for 72 h. (**E**) The viability of HK-2 cells treated with bupivacaine at 1 mM for 24 h. The viability of SKOV-3 (**F**) and PC-3 (**G**) were treated with bupivacaine (100 μM and 1 mM) plus anti-cancer drug taxol (100 nM) for 24 h. Data are presented as mean ± SD (n = 5). *P < 0.05; **P < 0.01; ***P < 0.001.

Bupivacaine at 1 mM decreased cell viability in both cell lines. Statistically significant effects were not observed at lower concentrations. A greater degree of cytotoxicity was exhibited when the treatment duration was 72 hours (Fig. 1A–D). Potentially different cytotoxic profiles between healthy and cancer cells to bupivacaine treatment were also explored. For this purpose, healthy human renal tubular epithelial (HK-2) cells were utilized. Interestingly, the change of cell viability in HK-2 was found to be not as significant as cancer cells after being treated with bupivacaine for 24 hours (Fig. 1E). This indicates that cancer cells, which are metabolically more active than their healthy equivalents, are more susceptible to bupivacaine’s cytotoxic properties. The synergetic effect of bupivacaine with chemotherapy agent taxol was also noted in both cell lines. Bupivacaine potentiated the toxic effects of taxol following 24 hours treatment. At doses of 100 μM or 1 mM, bupivacaine augmented the cytotoxicity of taxol at a dose of 100 nM (Fig. 1F,G).

### Bupivacaine on cancer cell apoptosis

**Figure 2:**
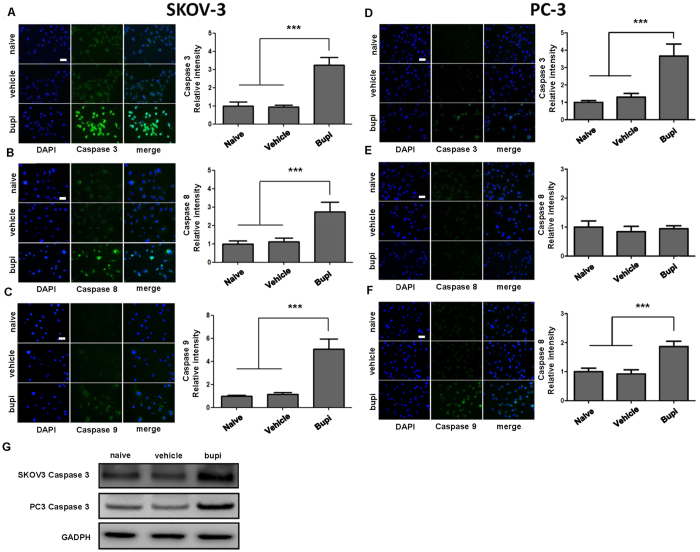
Bupivacaine changed cleaved-caspase expression in both ovarian cancer (SKOV-3) and prostate cancer (PC-3) cells. Cells were treated with 1 mM bupivacaine (Bupi) for 24 h followed by immunostaining and western blot. (**A**) Caspase 3, (**B**) Caspase 8, (**C**) Caspase 9 expression in SKOV-3 cells. (**D**) Caspase 3, (**E**) Caspase 8, (**F**) Caspase 9 expression in PC-3 cells. (**G**) Cleaved caspase 3 expression in SKOV-3 and PC-3 by western blot. Data are presented as mean ± SD. (n = 4). ***P < 0.001. Scale bar = 50 μm.

**Figure 3:**
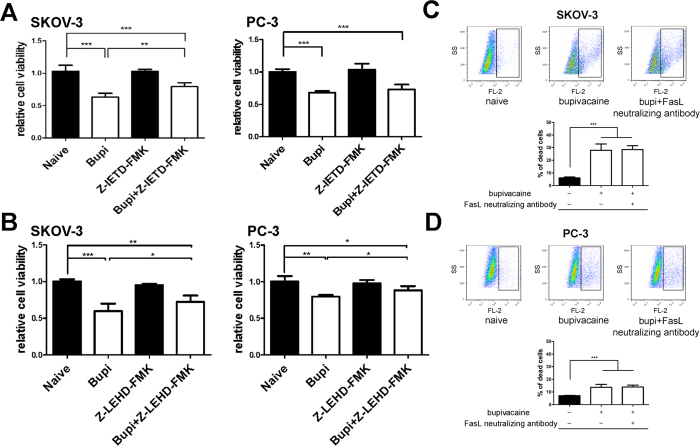
Effects of caspase 8 and 9 inhibition or Fas ligand neutralizing antibody on bupivacaine induced of ovarian cancer (SKOV-3) and prostate cancer (PC-3) cell death. Cells were pre-treated with 20 μM either caspase 8 inhibitor (Z-IETD-FMK) or caspase 9 inhibitor (Z-LEHD-FMK) 4 h before being superposed with 1 mM bupivacaine for 24 h. (**A**) Cell survival of caspase 8 inhibition in SKOV-3 and PC-3 cells. (**B**) Cell survival caspase 9 inhibition in SKOV-3 and PC-3 cells. Percentage of dead cells by the treatment of 500 ng/ml anti-Fas antibody (human, neutralizing, clone ZB4) in (**C**) SKOV-3 cells and (**D**) PC-3 cells. Data are presented as mean ± SD (n = 5). *P < 0.05; **P < 0.01; ***p < 0.001.

Caspase 3, 8 and 9 were activated in SKOV-3 following 1 mM bupivacaine treatment at 24 hours (Fig. 2A–C), with caspases 3 and 9 being cleaved in PC-3 (Fig. 2D–F). Cleaved caspase 3 expression through western blot were both elevated in two cancer cell lines after the treatment of 1mM bupivacaine (Fig. 2G). Caspase 9 inhibitor partially reversed bupivacaine induced SKOV-3 and PC-3 cell death (Fig. 3B), whilst Caspase 8 inhibition was effective in SKOV-3 only (Fig. 3A). Cytotoxicity was independent from FAS receptor activity as FAS ligand neutralization antibody treatment did not yield significant results as detected by flow cytometry (Fig. 3C,D).

### Bupivacaine on cancer cell proliferation and ROS production

**Figure 4:**
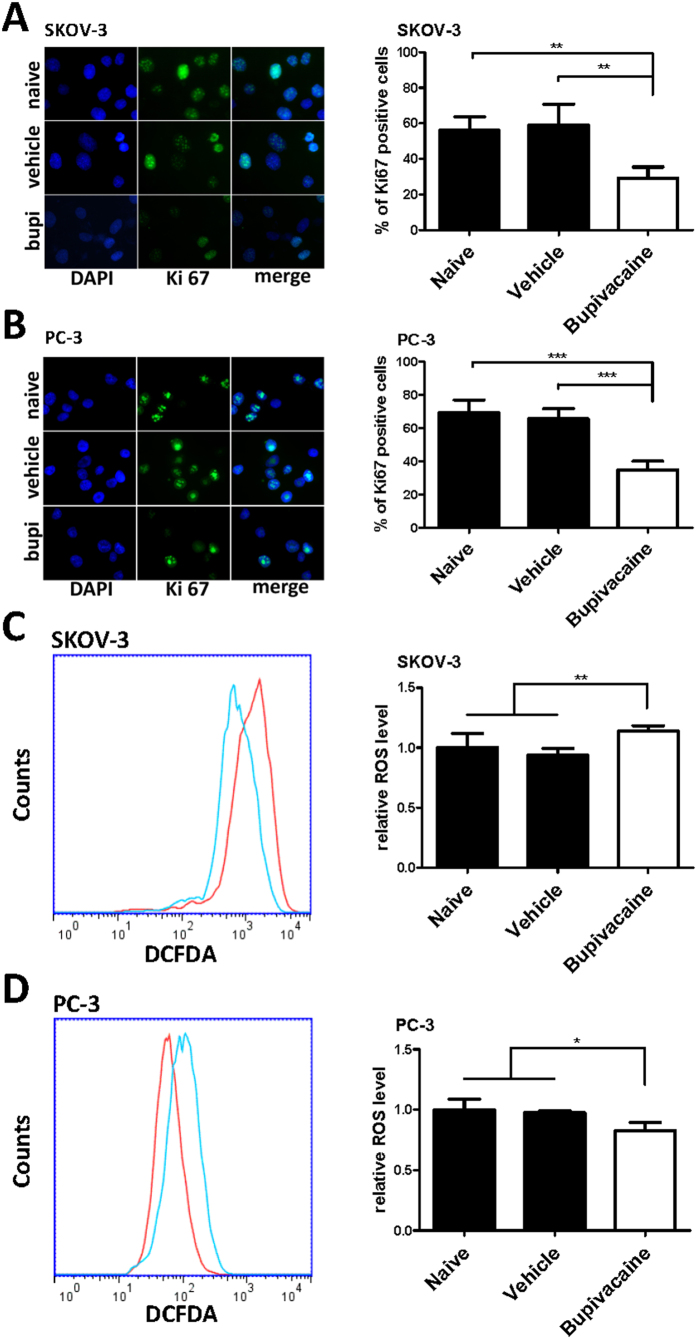
Bupivacaine treatment reduced cell proliferation and modulated oxidative stress of both ovarian cancer (SKOV-3) and prostate cancer (PC-3) cells. Cells were treated with 1 mM bupivacaine (Bupi) for 24 h for proliferation study, and 4 h treatment for reactive oxygen species (ROS) detection. (**A**) Ki-67 expression in (**A**) SKOV-3 cells and (**B**) PC-3 cells. (**C**) ROS generation in (**C**) SKOV-3 cells and (**D**) PC-3 cells. Data are presented as mean ± SD (n = 4). *P < 0.05; **P < 0.01; ***P < 0.001.

Bupivacaine treatment (1 mM) for 24 hours induced a statistically significant reduction in Ki-67 positive cells when compared to control groups in SKOV-3 (57.3% versus 30.1%, p < 0.01) and PC-3 cells (68.4% versus 36.7%, p < 0.001) (Fig. 4A,B). Bupivacaine induced a statistically significant increase in ROS generation in SKOV-3 (Fig. 4C), whereas ROS level decreased in PC-3 cells (Fig. 4D).

### Bupivacaine on cancer cell migration

**Figure 5:**
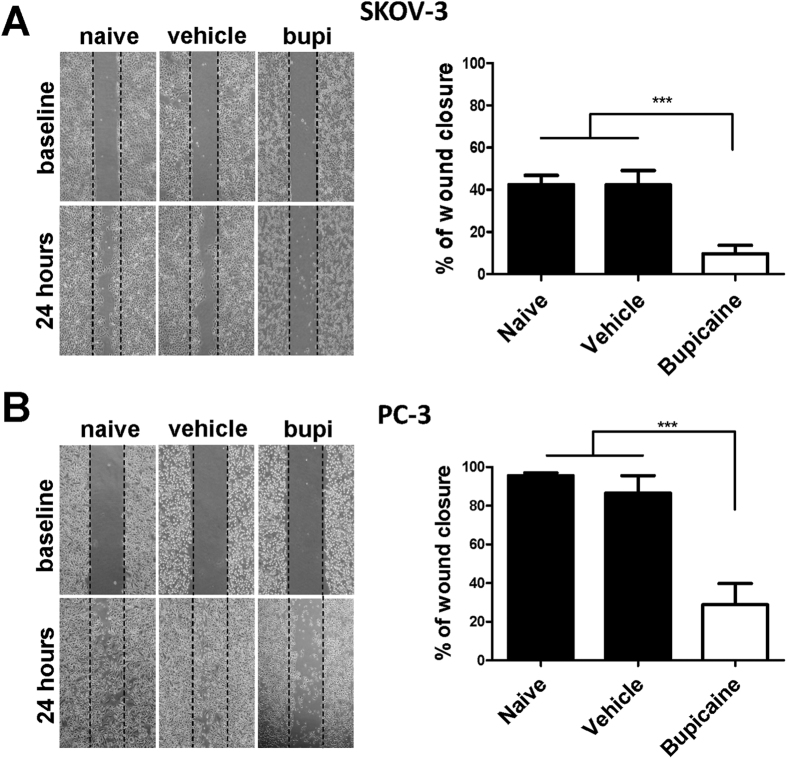
Bupivacaine treatment inhibited cell migration of both ovarian cancer (SKOV-3) and prostate cancer (PC-3) cells. Scratched monolayer cells were treated with 1 mM bupivacaine for 24 h. Degree of wound healing was calculated as (initial wound area − remaining wound area)/initial wound area. (**A**) Bupivacaine suppressed wound closure in SKOV-3 cells. (**B**) Bupivacaine suppressed wound closure in PC-3 cells. All values are presented as mean ± SD (n = 4); ***P < 0.001.

Wound healing assay was utilized to investigate the effects of bupivacaine on the migration potential of both cell lines. Following 24 hour bupivacaine treatment, fewer cells migrated towards the scratch midline in 1 mM bupivacaine treated group when compared to the control groups (Fig. 5A,B). Bupivacaine reduced migration potential of PC-3 cells by up to 60% (Fig. 5B). Lower concentrations of bupivacaine did not impact on cancer cell migration (data not shown).

### Bupivacaine on GSK-3β expression

**Figure 6:**
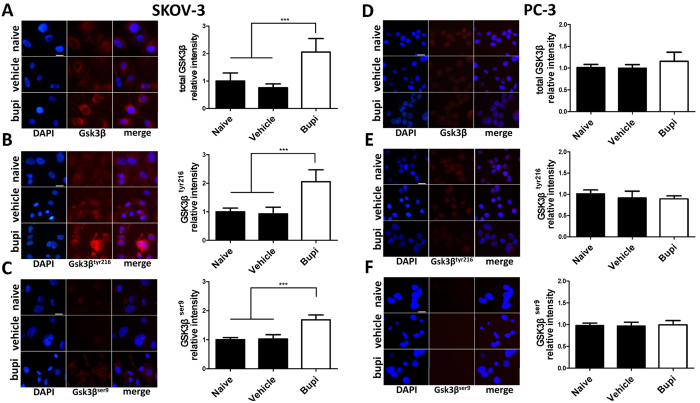
Bupivacaine increased GSK-3β, pGSK-3β^tyr216^ and pGSK-3β^ser9^ activation in ovarian cancer (SKOV-3) but not prostate cancer (PC-3) cells. Cells were treated with 1 mM bupivacaine for 24 h followed by immunostaining. (**A**) Total GSK-3β, (**B**) pGSK-3β^tyr216^, and (**C**) pGSK-3β^ser9^ expression in SKOV-3 cells. (**D**) Total GSK-3β, (**E**) pGSK-3β^tyr216^ and (**F**) pGSK-3β^ser9^ expression in PC-3 cells. Data are presented by mean ± SD (n = 4). ***P < 0.001. Scale bar = 50 μm.

Varying levels of increased expression in total GSK-3β, pGSK-3β^tyr216^ and pGSK-3β^ser9^ were demonstrated using immunofluorescence. In SKOV-3 cells, baseline levels of total GSK-3β and pGSK-3β^tyr216^ were similar and almost doubled following bupivacaine treatment when compared with control (Fig. 6A,B). The baseline level and elevation of pGSK-3β^ser9^ was relatively lower (Fig. 6C). With reference to the PC-3 cell line following bupivacaine treatment, similar basal expression levels of GSK-3β, pGSK-3β^tyr216^ and pGSK-3β^ser9^ were observed and these were not statistically significant (Fig. 6D–F).

### GSK-3 inhibition on bupivacaine induced SKOV-3 cell death

**Figure 7:**
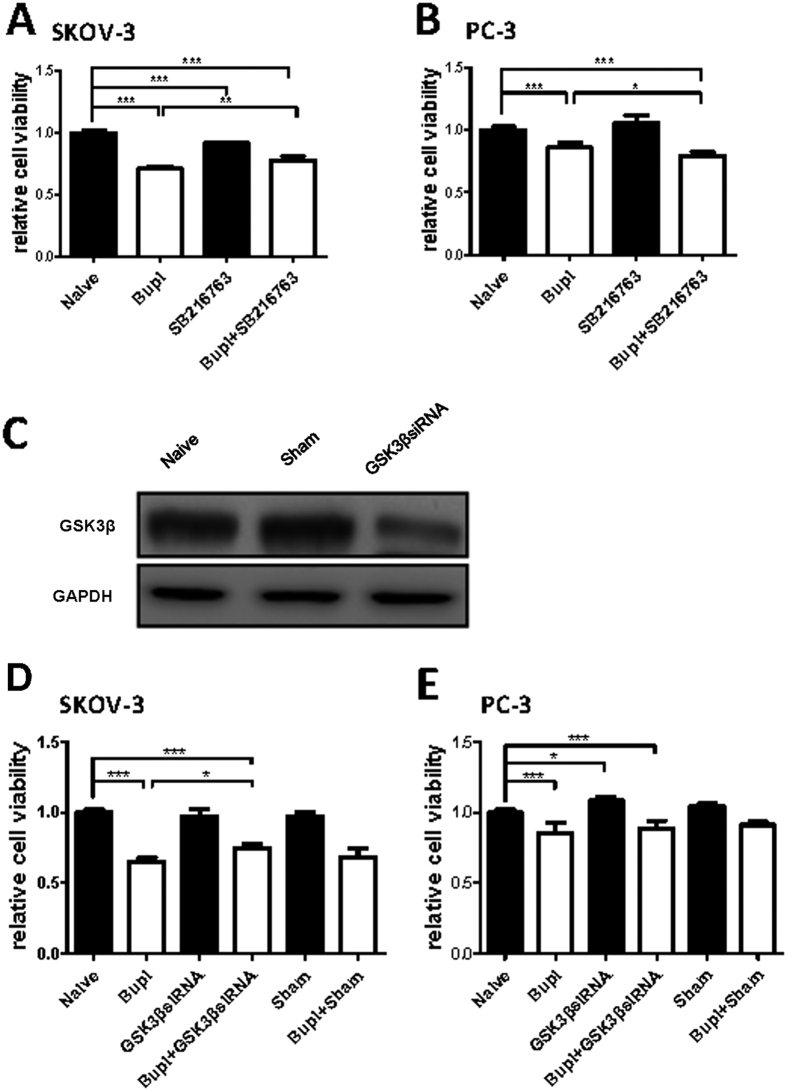
GSK-3 inhibitor (SB216763) or GSK-3β siRNA alleviated bupivacaine induced cell death in ovarian cancer (SKOV-3) but not prostate cancer (PC-3) cells. Cells were treated with 1 mM bupivacaine and 20 μM GSK-3 inhibitor for 24 h. The gene knockdown cells were treated with 1 mM bupivacaine for 24 h. Cell survival of GSK-3 inhibition in (**A**) SKOV-3 and (**B**) PC-3 cells. (**C**) Total GSK-3β expression by western blot. Cell survival of GSK-3β siRNA treatment in (**D**) SKOV-3 and (**E**) PC-3 cells. Data are presented as mean ± SD (n = 5). *P < 0.05; **P < 0.01; ***P < 0.001.

**Figure 8:**
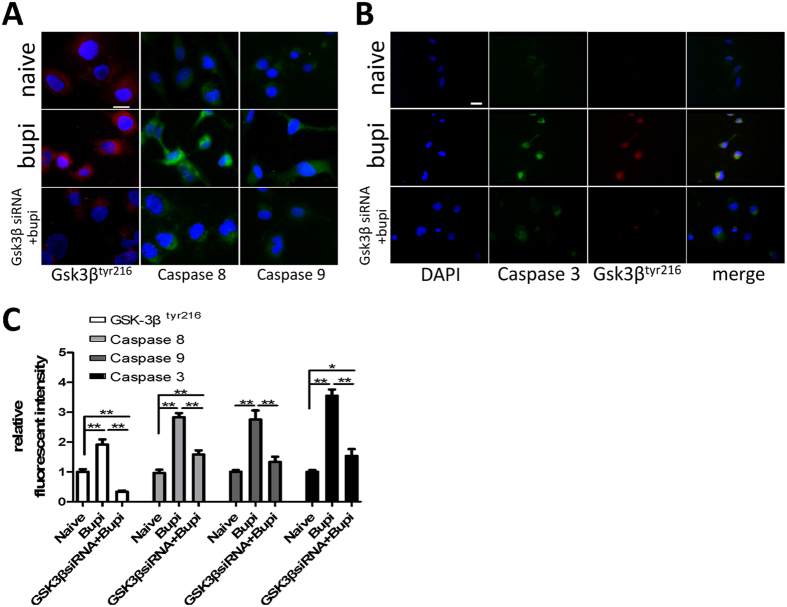
GSK-3β siRNA decreased the expression of caspase 3, 8, 9 and pGSK-3β^tyr216^. SKOV-3 cells transfected with siRNA were treated with 1 mM bupivacaine for 24 h. (**A**) pGSK-3β^tyr216^ (red) and caspase 8, 9 (green) expression in SKOV-3. (**B**) pGSK-3β^tyr216^ (red) and caspase 3 (green) co-expression. (**C**) The fluorescent intensity analysis of pGSK-3β^tyr216^ and cleaved caspase 3, 8 and 9. Data are presented as mean ± SD (n = 5). *P < 0.01; **P < 0.001. Scale bar = 50 μm.

GSK-3 pharmacological inhibitor SB-216763 and GSK-3β siRNA (gene knockdown) were used to examine potential GSK-3β activity in bupivacaine induced cell death. Total GSK-3β expression was almost abolished following GSK-3β siRNA treatment through western blot (Fig. 7C). Inhibition and gene knockdown both reduced the cytotoxic effects of bupivacaine induced SKOV-3 cell death (Fig. 7A,D). These reagents themselves did not have an effect in PC-3 cells (Fig. 7B,E). Following GSK gene knockdown, decreased caspase 3, 8, 9 cleavage and pGSK-3β^tyr216^ activity was observed (Fig. 8A–C).

## Discussion

**Figure 9:**
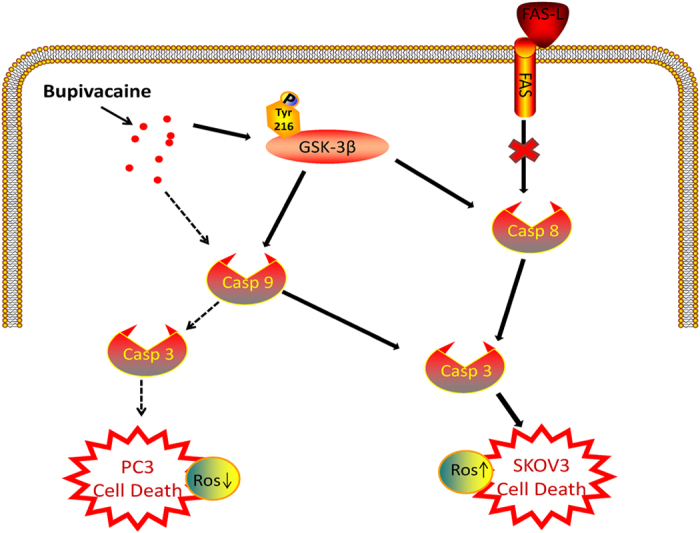
The proposed molecular mechanisms for bupivacaine induced cancer cell death. Bupivacaine induced ovarian cancer (SKOV3) cell death was associated with the activation of caspase 8 and 9, while only cleaved caspase 8 involved in prostate cancer cell death. The death pathway induced by bupivacaine is not linked to FAS receptor. The phosphorylation of GSK-3β on tyr 216 residue promote the sensitivity of ovarian cancer cells to bupivacaine induced cytotoxicity. Dashed line: death pathway for prostate cancer, solid line: death pathway for ovarain cancer. FAS-L = FAS ligand; GSK-3β = Glycogen synthase kinase-3β.

This study indicates that bupivacaine possesses cytotoxic, anti-proliferative and anti-metastatic properties in both ovarian and prostate cancer cell lines. Following 24 hours treatment, 1mM bupivacaine induced similar levels of cytotoxicity in both cell lines. Cell death was more pronounced after treatment for 72 hours. This is contrasted by the lack of statistical significant cell death exhibited by HK-2 cells when treated with 1mM bupivacaine. This may indicate different cell lines (cancerous vs non-cancerous) have varying levels of sensitivity to bupivacaine. Furthermore, an synergistic effect was observed when bupivacaine was combined with the chemotherapy agent taxol. Data is limited on potential mechanisms, but as shown in (Fig. 9) the activation of the intrinsic and extrinsic apoptotic pathways in conjunction with the active form of GSK-3β are likely to be involved (Fig. 9).

Data indicates there is an association between changes in cellular metabolism and the rate of cellular proliferation in cancer cells^16^. Beitner *et al.* reported that LA reduced melanoma cells glycolysis and ATP levels by downregulating two allosteric stimulatory signal molecules^17^. Lucchinetti *et al.* suggested that LA inhibited mesenchymal stem cells (MSC) proliferation^18^. Ki-67, a key marker of proliferation^19^, was used in our study to examine the effects of LA on cellular proliferation. Our results show decreased Ki67 expression in both SKOV-3 and PC-3 cell lines treated with 1 mM bupivacaine.

Lucchinetti *et al.*^18^ reported that 100 μM ropivacaine significantly inhibited mesenchymal stem cell migration as measured by wound healing assay. Our study demonstrates that 1 mM bupivacaine inhibits the migration potential of SKOV-3 and PC-3. Greater inhibition was observed in PC-3. Data indicates that prostate cancer exhibits higher levels of metastatic potential^20^. This may suggest that the anti-migration properties of LA may have a greater impact on more invasive tumors.

Reactive oxygen species (ROS) activity was also determined in the current study. It has been reported that increased ROS levels suppresses breast cancer cell proliferation^21^, whilst opposite finding also exists is that antioxidants can inhibit liver cancer cell proliferation^22^. Our study demonstrates increased ROS levels in SKOV-3, whilst decreased ROS levels in PC-3. These results are in keeping with the contradictory role ROS activity appears to have in cancer cell growth/proliferation^21^,^22^ and requires further investigation.

Previous studies indicate that LA induced cancer cell death is caspase dependent^23^. Werdehausen *et al.*^24^ used gene modulation techniques and demonstrated that low concentrations of lidocaine induced Jurkat cell death *via* the intrinsic apoptotic pathway. We found that caspase 3, 8 and 9 were activated in SKOV-3 following 1 mM bupivacaine treatment while caspase 3 and 9 were cleaved in PC-3. Furthermore, partial cell death inhibition was observed with caspase 9 inhibition in both cell lines. Caspase 8 inhibition was only effective in SKOV-3. It has been noticed that mutations in tumor suppressor genes; such as BRCA1 in ovarian cancer, have a significant function in DNA damaging related apoptosis in cancer chemotherapy^25^. Local anesthetics have also demonstrated to be potent DNA damaging agents. Kim *et al.* demonstrated that dibucaine induced DNA fragmentation and chromatin condensation in neuroblastoma cells^26^. It is to be determined if genomic defects increase the cytotoxic profile of bupivacaine in ovarian and prostate cancer.

The caspase cascade involves numerous cellular processes; of which FAS ligand activity is implicated. This study indicates FAS ligand receptor activity is not involved in bupivacaine interactions with SKOV-3 or PC-3. It has been reported that caspase 8 activation not only occurs via FAS/death receptor ligand^27^, but also via FAS ligand independent caspase 8 induced cell death^28^. This suggests different cancer cells exhibit varying biological profiles which influence molecular signal transduction processes involved in growth, development and death.

Glycogen synthase kinase-3β (GSK-3β) is a serine/threonine kinase which is implicated in numerous cell functions including cell differentiation, proliferation and apoptosis. GSK-3β leads cell apoptosis via the interaction with proapoptotic transcription factor p53 as its regulatory protein, and it also cause apoptosis by inducing mitochondrial injury and the caspase cascade^29^,^30^. Phosphorylation at its tyrosine residue (tyr-216) constitutes its active form whereas serine residue (ser-9) phosphorylation is its inactive form^31^. The role of GSK3β in tumor development is controversial. Previous studies have shown that GSK3β impaired tumor growth in several cancer cell lines^32^,^33^. However, Cao *et al.*^34^ reported that the suppression of kinase inactive form GSK3β^ser9^ promoted ovarian cancer development, which indicated GSK3β is also necessary for tumor survival. Furthermore, another study showed that the suppression of Src-GSK3β axis could be a new target to treat prostate cancer^35^. GSK3β interactions with chemotherapy agents are complicated. There is increasing evidence which indicates that GSK3β activity modulates the effectiveness of chemotherapy on cancer cells. Downregulation of GSK3β expression level conferred resistance of ovarian cancer cells from cisplatin treatment^36^. In hepatoblastoma cell lines, GSK3β inhibition by pharmacological or gene knockdown/mutant techniques limited anti-cancer drug induced apoptosis^37^. Consistent with these findings, our study demonstrates GSK3β expression is essential for bupivacaine induced cell death. Total GSK3β, pGSK-3β^ser9^ and pGSK-3β^tyr216^ were all elevated in SKOV-3 cells following 24 hours of treatment with 1 mM bupivacaine. Greater levels of expression were observed in pGSK-3β^tyr216^, the active form of GSK3β, when compared with the inactive form pGSK-3β^ser9^. Previous reports have primarily focused on GSK-3β^ser9^ or GSK-3β^tyr216^ expression in isolation. Our findings indicate that whilst both were activated, an overall increase in the expression of GSK-3β is observed. Statistically significant changes in expression of GSK3β,pGSK-3β^ser9^ and pGSK-3β^tyr216^ were not observed in the PC-3 cell line. This indicates that bupivacaine induced prostate cancer cell death is unlikely to involve GSK3β activity.

To further examine GSK-3β activity and the relative expression of its residues, GSK3β inhibition demonstrated the partial suppression of cell death in bupivacaine treated SKOV-3 cells. This suggests GSK3β is pro-apoptotic in bupivacaine induced cell death. To investigate potential interactions between GSK3β and caspase activity in apoptosis, we demonstrated that caspase 3, 8 and 9 were down regulated following GSK3β siRNA treatment, which reaffirmed the hypothesis that GSK3β deactivation in the SKOV-3 confers resistance to bupivacaine induced cell death.

In summary, our findings suggest that bupivacaine has direct ‘anti-cancer’ properties *in vitro*. However, our work is not without limitations. Firstly, these *in vitro* experiments do not fully replicate an *in vivo* or clinical environment and thus warrant further study. Secondly, the concentrations of bupivacaine (up to 1 mM) tested in this study may not be applicable in particular clinical contexts. LA concentrations vary according to their mode of delivery. The concentration of LA on direct infiltration has been reported to reach 500 μM. It can be even higher when administered topically^38^. An extension of this even though this is beyond the scope of this study is to acknowledge that the cancer microenvironment is complex and this in itself is an important determinant in tumor growth and metastasis. It has been shown that in the presence of TNF-alpha, a low dose of LA suppressed TNF-alpha induced ICAM-1 phosphorylation which is associated with LA anti-migration properties^14^. Finally, regional anesthesia combined general anesthesia with inhalational agents are often used in cancer patients^39^,^40^. The potential interaction between local anesthetics and inhalational anesthetics on cancer cell biology has not been investigated in this study. Interestingly, the survival rate from cancer recurrence is much higher with regional anesthesia combined with general anesthesia than with general anesthesia alone^9^,^41^. These observations are discussed in recently published literature^42^,^43^. They indicate that inhalational anesthetics (e.g. isoflurane) may promote cancer cell malignancy *in vitro.* This would require further investigation to establish if they hold true *in vivo* and ultimately in clinical practice. Nevertheless, the data reported here clearly demonstrates that the local anesthetic bupivacaine can directly “kill” cancer cells through mechanisms not explored before in this context, which is likely to stimulate further research and increase the call for more clinical trials to be conducted.

## Materials and Methods

### Cell Culture

Human ovarian carcinoma (SKOV-3), prostate carcinoma (PC-3) and human proximal tubular cell (HK-2) lines (all purchased from European cell culture collection, Salisbury, UK) were used for this study. SKOV-3 were cultured in McCoy’s 5A medium (Sigma Aldrich, St. Louis, USA) and PC-3 and HK-2 were cultured in RPMI-1640 medium (Sigma Aldrich, St. Louis, USA). Culture mediums were supplemented with 10% newborn calf serum (HyClone, Auckland, New Zealand) with 1% L-glutamine and 1% penicillin-streptomycin (Sigma Aldrich, St. Louis, USA). Cells were maintained at 37 °C under a humidified atmosphere of 5% CO_2_ and 95% air in an air jacket incubator (Triple Red, Buckinghamshire, UK). All cells were cultured in 24 wells plate and used for experiment when reach 70–80% confluence.

Cells were cultured with bupivacaine at concentrations ranging from 1 μM to 1 mM for 24 or 72 hours. Other cohort cultures were treated with 100 μM/1 mM bupivacaine or together with 100 nM chemotherapy drug taxol (Sigma Aldrich, St. Louis, USA) or other inhibitors for certain period of time for further experiments described below. All treated cultures and the appropriate controls were analyzed for various end-points with the methods described below.

### FAS, Caspase-8, caspase-9 inhibition

Cells were pre-treated for 30 minutes with FAS neutralizing antibody ZB4 (Millipore, Billerica, MA, USA) at a final concentration of 500 ng/ml as previously reported^44^. Other cohort cells were pre-treated with either 20 μM caspase 8 inhibitor Z-IETD-FMK or caspase 9 inhibitor Z-LEHD-FMK (both purchased from R&D Systems Minneapolis, USA) 4 hours followed with 1 mM bupivacaine for 24 hours.

### GSK-3β inhibition by inhibitor or siRNA

GSK-3 inhibitor (20 μM) (SB-216763, EMD Millipore Corp, Billerica, USA) superposed with 1 mM bupivacaine were given to cultures for 24 hours. Other cells were cultured with siRNA (sc-35527, Santa Cruz Biotechnology, CA, USA) targeting GSK-3β or unspecific scramble siRNA dissolved in siRNA suspension buffer supplemented with lipofectamine (Invitrogen, Paisley, UK) to a final concentration of 20 nM for 6 h. Afterwards, cells were superposed with 1 mM bupivacaine for 24 hours.

### Cell viability and death measurement

Cell viability was assessed by 3-(4,5-dimethylthiazol-2-yl)-2,5-diphenyltetrazolium bromide (MTT) (EMD Chemicals, San Diego, CA) assay reported elsewhere^45^. MTT was dissolved in Opti-MEM (Gibco, Paisley, UK) to form 0.5 mg/ml working solution. Culture medium was removed after the incubation with or without indicated concentrations of LA and taxol, then 500 μL MTT working solution was added into each well and incubated for 4 hours. Following this, supernatants were aspirated and 500 μL DMSO (Fisher Scientific, Leicestershire, UK) was added to dissolve the formazan crystals. In 96 well plates, absorbance was measured at 595 nm using micro plate reader analysis (Dynex technologies, Chantilly, VA, USA). Cell viability relative to the control was calculated and expressed as relative to control.

For further clarification, Propidium iodide (PI) (Sigma Aldrich, St. Louis, USA) staining was used to examine cell death as described previously^46^. Cells were harvested in a FACS tube and washed twice before re-suspension in FACS buffer. PI was added to make the final concentration to 1 μg/ml and incubated in dark for 5 min. Cells were not washed then PI fluorescence was detected using flow cytometry (FL-2 channel). Single color positive cells were defined as dead cells.

### Immunofluorescence

After treatments, SKOV-3 or PC-3 cells were fixed in 4% paraformaldehyde, then blocked with donkey serum in PBST (Sigma Aldrich, St. Louis, USA) for 1 hour followed by the primary antibody: rabbit anti-cleaved caspase 3, 8 or 9 (Abcam, Cambridge, UK), mouse anti-caspase 3 (Abcam, Cambridge, UK), mouse anti-Ki-67 (Dako, Cambridge, UK), rabbit anti-GSK3β, pGSK-3β^ser9^ or pGSK-3β^tyr216^ (Abcam, Cambridge, UK) (1:200) in PBST overnight at 4 °C followed by FITC or Rhodamine conjugated secondary antibody (Millipore, Watford, UK) (1:400). The slides were counterstained with nuclear dye 4′,6-diamidino-2-phenylindole (DAPI) and mounted with VECTASHIELD Mounting Medium (Vector Laboratories, Burlingame, CA) and then examined using an Olympus BX4 microscope (Watford, UK). Immunofluorescence was quantified using ImageJ (National Institutes of Health, Bethesda, MD, USA). Ten representative regions per section were randomly selected by an assessor blinded to the treatment groups. Values were then calculated and expressed as relative to control.

### Reactive oxygen species measurement

Intracellular reactive oxygen species (ROS) generation was detected by flow cytometry with 5- (and -6)-carboxy-2′,7′-dichlorofluorescein diacetate (DCFH-DA) (Sigma Aldrich, St. Louis, USA) probe. Briefly, cells were washed twice with pre-warmed Dulbecco’s phosphate-buffered saline (DPBS) (Sigma Aldrich, St. Louis, USA). 10 μM DCFH-DA was added into FACS tube to stain cells in dark for 30 min at 37 °C. Immunofluorescence intensity was acquired and analyzed using flow cytometry (FACSCalibur; Becton Dickinson, Sunnyvale, CA). Each trial had at least 10,000 gated events.

### Wound healing assay

Capability of migration was evaluated by wound healing assay reported previously^47^. Briefly, cells were cultured in a 60 mm petri dish to form a confluent monolayer. Then, a wound was scratched on this monolayer by 1 ml pipet tip and washed twice with culture media. Before taking each image, a mark was made at the bottom of the dish to make sure that all the images were taken at the same site. After another 24 hours incubation with or without various concentrations of bupivacaine, the second image was taken at the same site. The wound healing status was compared by Image-Pro Plus software (Media Cybernetics, USA) based on these images. The results were presented as (initial wound area − remaining wound area)/(initial wound area).

### Western blot

Cell samples were homogenized in lysis buffer. Cell lysates were centrifuged with the supernatant being collected. Then protein concentration was quantified in the supernatant by Bradford protein assay (Bio-Rad, United Kingdom). Protein extracts (40 μg per sample) were heated, denatured, and loaded on a NuPAGE 4 to 12% Bis-Tris gel (Invitrogen, USA) for electrophoresis and transferred to a polyvinylidene difluoride membrane. The membrane was blocked with 5% non-fat milk for 1 h at room temperature and then probed with rabbit anti-cleaved caspase-3 and mouse anti-GSK-3β (1:1000; Santa Cruz, USA) primary antibody in tris-buffered saline and Tween-20 overnight at 4 °C, followed by the application of goat anti-rabbit or goat anti-mouse horseradish peroxidase-conjugated secondary antibody for 1 h at room temperature. The loading control was protein GAPDH (1:10,000; Millipore, USA). The blots were detected with enhanced chemiluminescence system (Santa Cruz, USA) and analyzed with GeneSnap (Syngene, United Kingdom). Protein band intensity was normalized with GAPDH and expressed as a ratio of control.

### Statistical analysis

All data were expressed as Mean ± SD. Data were analyzed by one-way analysis of variance, followed by the post hoc Student–Newman–Keuls test (GraphPad Prism 5.0 software, San Diego, CA) for comparison with Bonferroni corrections. A p value < 0.05 was considered to be statistically significant.

## Additional Information

**How to cite this article**: Xuan, W. *et al.* Local anesthetic bupivacaine induced ovarian and prostate cancer apoptotic cell death and underlying mechanisms *in vitro*. *Sci. Rep.* **6**, 26277; doi: 10.1038/srep26277 (2016).
